# The intrinsic and microenvironmental features of diffuse midline glioma: Implications for the development of effective immunotherapeutic treatment strategies

**DOI:** 10.1093/neuonc/noac117

**Published:** 2022-04-27

**Authors:** Mika L Persson, Alicia M Douglas, Frank Alvaro, Pouya Faridi, Martin R Larsen, Marta M Alonso, Nicholas A Vitanza, Matthew D Dun

**Affiliations:** Cancer Signalling Research Group, School of Biomedical Sciences and Pharmacy, College of Health, Medicine and Wellbeing, University of Newcastle, Callaghan, New South Wales, Australia; Precision Medicine Program, Hunter Medical Research Institute, New Lambton Heights, New South Wales, Australia; Cancer Signalling Research Group, School of Biomedical Sciences and Pharmacy, College of Health, Medicine and Wellbeing, University of Newcastle, Callaghan, New South Wales, Australia; Precision Medicine Program, Hunter Medical Research Institute, New Lambton Heights, New South Wales, Australia; Cancer Signalling Research Group, School of Biomedical Sciences and Pharmacy, College of Health, Medicine and Wellbeing, University of Newcastle, Callaghan, New South Wales, Australia; Precision Medicine Program, Hunter Medical Research Institute, New Lambton Heights, New South Wales, Australia; John Hunter Children’s Hospital, New Lambton Heights, New South Wales, Australia; Department of Medicine, School of Clinical Sciences, Monash University, Melbourne, Victoria, Australia; Department of Molecular Biology and Biochemistry, Protein Research Group, University of Southern Denmark, Odense, Denmark; Department of Pediatrics, University Hospital of Navarra, Pamplona, Spain; Program in Solid Tumors and Biomarkers, Foundation for Applied Medical Research (CIMA), Pamplona, Spain; Ben Towne Center for Childhood Cancer Research, Seattle Children’s Research Institute, Seattle, Washington, USA; Department of Pediatrics, Seattle Children’s Hospital, University of Washington, Seattle, Washington, USA; Cancer Signalling Research Group, School of Biomedical Sciences and Pharmacy, College of Health, Medicine and Wellbeing, University of Newcastle, Callaghan, New South Wales, Australia; Precision Medicine Program, Hunter Medical Research Institute, New Lambton Heights, New South Wales, Australia

**Keywords:** diffuse intrinsic pontine glioma (DIPG), diffuse midline glioma (DMG), immuno-oncology, immunotherapy, pediatric high-grade glioma (HGG)

## Abstract

Diffuse midline glioma (DMG), including those of the brainstem (diffuse intrinsic pontine glioma), are pediatric tumors of the central nervous system (CNS). Recognized as the most lethal of all childhood cancers, palliative radiotherapy remains the only proven treatment option, however, even for those that respond, survival is only temporarily extended. DMG harbor an immunologically “cold” tumor microenvironment (TME) with few infiltrating immune cells. The mechanisms underpinning the cold TME are not well understood. Low expression levels of immune checkpoint proteins, including PD-1, PD-L1, and CTLA-4, are recurring features of DMG and likely contribute to the lack of response to immune checkpoint inhibitors (ICIs). The unique epigenetic signatures (including stem cell-like methylation patterns), a low tumor mutational burden, and recurring somatic mutations (H3K27M, *TP53*, *ACVR1*, *MYC*, and *PIK3CA*), possibly play a role in the reduced efficacy of traditional immunotherapies. Therefore, to circumvent the lack of efficacy thus far seen for the use of ICIs, adoptive cell transfer (including chimeric antigen receptor T cells) and the use of oncolytic viruses, are currently being evaluated for the treatment of DMG. It remains an absolute imperative that we improve our understanding of DMG’s intrinsic and TME features if patients are to realize the potential benefits offered by these sophisticated treatments. Herein, we summarize the limitations of immunotherapeutic approaches, highlight the emerging safety and clinical efficacy shown for sophisticated cell-based therapies, as well as the evolving knowledge underpinning the DMG-immune axis, to guide the development of immunotherapies that we hope will improve outcomes.

Diffuse midline glioma (DMG) is a pediatric and adolescent high-grade glioma (HGG) originating along the midline structures of the brain, including the pons (diffuse intrinsic pontine glioma [DIPG]), and arises from the malignant transformation of stem cells of the oligodendroglial lineage.^1^ Approximately 300-350 cases are diagnosed each year in the United States, which constitutes 10%-20% of all pediatric cancers diagnosed in the central nervous system (CNS). Overall survival (OS) for children and adolescents diagnosed with DIPG is 9-11 months, with <10% surviving 2-year post-diagnosis.^2–5^ The high mortality rate makes DMG the leading cause of death in children with CNS tumors.

Global hypomethylation of histone H3K27 is seen in every case of DMG. Driven by recurring somatic mutations in H3 genes (including *HIST1H3B/C* (H3.1K27M) or *H3F3A* (H3.3K27M), subtyped “H3 mutant”) or through overexpression of the Enhancer of Zest Homologs Inhibitory Protein (EZHIP) in patients harboring wild-type H3. These alterations inhibit the catalysis of H3K27 trimethylation by the Polycomb repressive complex 2 (PRC2).^6–9^ The 5th edition of the World Health Organization (WHO) Classification of Central Nervous System (CNS) Tumors (published 2021), designates the malignancy “Diffuse midline glioma, H3K27-‘altered’” (reflective of the multiple underlying molecular and epigenetic alterations) and includes those of the brainstem (pons, midbrain, medulla oblongata) as well as tumors found in the thalamus and spine.

Unfortunately, chemotherapies have not prolonged survival for patients with DMG, and because of the delicate anatomical localities, significant resection is impossible, hence biopsies have been historically few. Therefore, most prior studies have not evaluated immune cell infiltration in DMG patient tumors.^10^ However, with advancing surgical techniques, biopsy has become more commonplace.^11^ Subsequent landmark molecular studies describing the recurring somatic features of the disease at diagnosis have encouraged precision medicine-based treatment strategies, however, again, the approach is yet to deliver improvements in patient survival.^12,13^ The standard of care treatment for DMG remains palliative radiotherapy which extends survival by only months for those that respond.^14^ In addition to radiation, the immunosuppressant corticosteroid, dexamethasone is almost always administered to decrease the neurological symptoms associated with peritumoral edema. Hence, there is a clear priority to develop new treatment strategies for children and young adults diagnosed with DMG.^15^

Since the approval of immune checkpoint inhibitors (ICI) for the treatment of melanoma, non–small-cell lung cancer (NSCLS), and Hodgkin lymphoma, immuno-oncology (IO)-based treatment approaches are being hailed as the next generation of cancer care. However, as it currently stands, this has not been the experience in aggressive CNS cancers. Given that biopsy has only recently become more commonplace, thus facilitating the collection of the necessary tissue to survey for potential immunotherapy targets, IO approaches in DMG lag behind that of more common cancer types. Here, we review the unique genomics and epigenetics of DMG, and discuss the locality of these tumors to outline the limitations and highlight opportunities for current and future IO treatment approaches. We also discuss successful strategies used in other cancer types and those currently in clinical trials for the treatment of DMG.

## Unique Characteristics of Diffuse Midline Glioma and Their Roles in Immuno-oncology Treatment Strategies

**Fig. 1:**
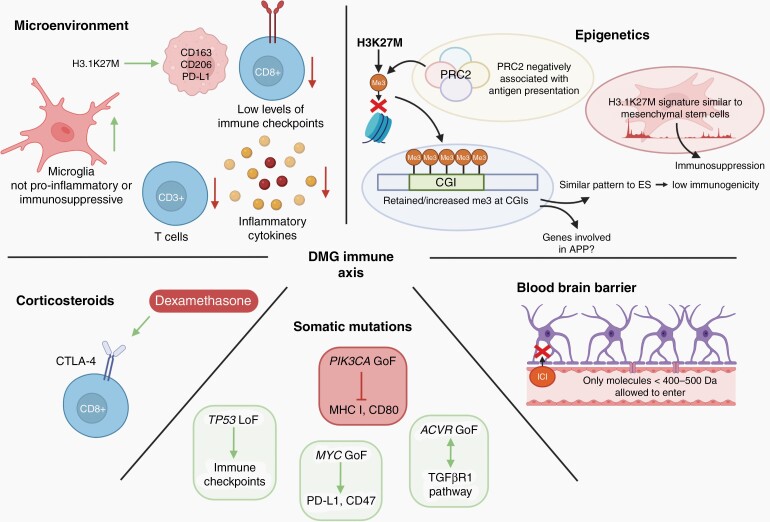
Contributors to the DMG-immune axis. The unique epigenetic signatures of H3K27M DMG are similar to both embryonic stem cells (ESC) and mesenchymal stem cells (MSC), known to have low immunogenicity and to be immunosuppressive, respectively. Additionally, PRC2 activity shows a negative relationship with antigen presentation. Several known somatic mutations in DMG are linked to immunosuppression. Corticosteroids drive the expression of CTLA-4. DMG lack T-cell infiltration, inflammatory cytokine expression, and immune checkpoint proteins, with increased immune-neutral microglia cell residency. H3.1K27M DMG promotes microglia with immunosuppressive markers. The BBB prevents most molecules (>400 to 500 Da) from entering the brain. Abbreviations: BBB, blood-brain barrier; DMG, diffuse midline glioma; PRC2, polycomb repressive complex 2; CGI, CpG islands.

IO strategies approved by the US Food and Drug Administration (FDA) have transformed the treatment of highly aggressive cancers, even in cases refractory to all other conventional approaches.^16^ Unfortunately, the use of ICIs has not provided any survival benefit, and in the case of pembrolizumab, a PD-1 (programmed cell death protein 1) inhibitor, even decreased median progression-free survival (PFS) in patients with DMG (discussed in the section “PD-1 and PD-L1”). Primary DMG and its tumor microenvironment (TME) display limited expression of PD-1/PD-L1 and little immune cell infiltration when compared to other HGG (such as glioblastoma [GBM]) and other low-grade glioma (LGG).^10,17^ This, together with minimal immune activation in the DMG microenvironment, is consistent with the cell of origin and the observations that cancer stem cells are not efficiently targeted for immune destruction by T cells.^10,18^ It is not clear what impact the immunologically “cold” TME plays in the gliomagenesis of DMG, however, emerging data describing various unique DMG characteristics may provide us with clues regarding the mechanisms driving avoidance of immune system surveillance (discussed in the sections “DMG Epigenetics,” “DMG-Associated Somatic Mutations,” and “Immunosuppression”) and highlight strategic opportunities which we may be able to harness in the development of new, effective IO strategies (Figure 1).

### DMG Epigenetics

Global hypomethylation of histone H3 at K27 is the hallmark of DMG. In normal tissues, H3K27 is either acetylated (H3K27ac), or mono- (H3K27me1), di- (H3K27me2), or trimethylated (H3K27me3). H3K27ac marks active enhancers, with recent reports also suggesting that H3K27me1 is associated with active transcription. However, the effects of monomethylation remain under discussion.^19^ In contrast, deposition of H3K27me2 and H3K27me3 marks are catalyzed by PRC2 and associated with transcriptional repression. Of note in DMG, substitution from lysine to methionine in residue 27 (H3K27M) prevents methylation not only of the mutant allele (H3K27M) but also throughout the remaining wild-type H3 alleles, analogous to H3-wild-type DMG where overexpression of EZHIP prevents PRC2 from catalyzing trimethylation.^7–9^ Thus, even though only 3%-17% of total histone H3.1 and H3.3 harbor the K27M mutation in a DMG cell, a global reduction of H3K27me2/me3 persists throughout (dominant negative).^8^ Loss of transcriptional silencing is strongly linked to the gliomagenesis of DMG, however, subtle differences in H3.1K27M, H3.3K27M, and EZHIP tumor subtypes are reported including co-occurring somatic mutations, locality, age at diagnosis, and median OS, with the latter suggesting these characteristics influence responses to radiotherapy and/or immunotherapy, and hence, time to relapse and death.^15,20^

The H3.1 subtype has been shown to have a gene signature more similar to “mesenchymal glioblastoma”.^20^ Interestingly, a mesenchymal phenotype has been associated with a more immunosuppressive microenvironment in NSCLC^21^ and melanoma,^22^ including increased expression of immune checkpoint proteins, less immune cell infiltration and less response toward ICI therapies.^21,22^ This suggests that the epigenetics of H3.1K27M DMGs might promote a more immunologically cold microenvironment (Figure 1).

Intriguingly, previous studies have shown that PRC2 expression is negatively associated with antigen presentation; inhibition of EZH2 (the catalytic subunit of PRC2) increases antigen presentation and T-cell infiltration.^23^ In H3K27M DMG, specific loci in the genome remain trimethylated, and chromatin at specific CpG islands (CGI) even shows increased trimethylation.^24^ This sustained methylation is essential for DMG cell survival as EZH2 inhibitors inhibit cell growth. Furthermore, similar trimethylation confined to CGIs is also seen in embryonic stem cells (ESCs).^25^ ESCs have a low expression of the major histocompatibility complex I (MHC I), making these primitive cells less visible to the immune system.^26^ Thus, it would be interesting to uncover if the sustained H3 methylation in DMG involves suppression of the antigen presentation pathway (APP) to possibly guide treatments that increase MHC I expression and thus increase the immunogenicity of the DMG tumor.

### DMG-Associated Somatic Mutations

DMG is known to have a low tumor mutational burden (TMB), and hence possibly harbor few neoantigens presented on their cell surface.^15^ A low TMB has been linked to decreased response to ICIs in several cancers; however, its use as a predictive marker is uncertain.^27^ Nevertheless, this implies that monotherapy ICI might not provide the increased DMG patient survival so desperately needed (further discussed in the section “Immune Checkpoint Inhibitors”).

The most common somatic mutations after H3K27M in DMG include mutations in *TP53*, *PDGFRA*, *ACVR1*, *MYC*, and members of the PI3K pathway.^15,28,29^ It is still unknown if any of these mutations impact the TME in DMG. Intriguingly, in several cancers, loss of function (LoF) mutations in *TP53* have been associated with an immunosuppressive microenvironment.^30,31^ Even though further studies are required to determine the mechanisms underpinning *TP53*-induced immunosuppression, current studies point to an increase in immune checkpoint protein expression possibly sensitizing the tumor to ICIs.^30,31^ This is yet to be observed/reported in DMG, however.^10^

Activating mutations in *ACVR1* are seen in 32% of all DMG^15,29^ and show crosstalk with the transforming growth factor-beta receptor 1 (TGFβR1) pathway.^32^ TGFβR1 is a receptor of TGF-β, an immunosuppressive cytokine. TGFβR1 inhibition has been shown to be less effective in H3.1K27M *ACVR1* G328V mutant DMG compared to H3.3K27M *ACVR1* wild-type DMG, suggesting that ACVR1 drives some of the effects of TGFβR1.^32^ Nevertheless, its implication in eventual immunosuppression is still unclear.

Mutations in the components of the phosphatidylinositol-4,5-bisphosphate 3-kinase (PI3K) signaling axis are recognized drivers of gliomagenesis in DMG.^15,29^ In pancreatic ductal adenocarcinoma (PDAC) knockout of *PIK3CA* (p110α catalytic subunit of PI3K signaling) led to T-cell infiltration and tumor clearance in a mouse model^33^; the lack of *PIK3CA* increased expression of MHC I and the T-cell co-stimulator CD80. Although confirmatory studies are required, this suggests that the activated PI3K pathway (seen in the majority of DMG) may also decrease the expression of MHC I and CD80 on tumor cells, making them invisible to the immune system. Additionally, inhibition of the PI3K pathway combined synergistically with anti-PD-1 therapies, further highlighting the pathway’s involvement in immunogenicity.^34^

The oncogene *MYC* is altered in 20% of DMG (H3.3K27M^+^).^35^ In T-cell acute lymphoblastic leukemia (T-ALL), *MYC* is shown to upregulate both PD-L1 and CD47, protecting the cells from T cells and macrophages, respectively,^36^ encouraging investigation as to whether targeting these mutations may enhance susceptibility to IO treatments in DMG.

Analysis of non-silent mutations in DMG has revealed significant intratumor heterogeneity.^37^ This diversity represents a relatively unexplored field, and provides a lens through which we may be able to better understand the aggressive treatment-resistant features of this cancer.^15^ Importantly, the heterogenous nature of DMG may portend avoidance of immune elimination through immune editing; some cells acquire mutations resulting in reduced immunogenicity enabling them to avoid elimination and allowing them to be maintained in a dormant state at equilibrium. The pressure from the adaptive immune system thus selects for better-equipped subclones, referred to as immunoselection. This begs the question as to whether immunotherapies that co-target several mutations/antigens are required to circumvent resistance.

### Immunosuppression

Control of peritumoral inflammation with corticosteroids, such as the commonly prescribed dexamethasone, may also contribute to immune evasion (Figure 1). Dexamethasone has been shown to upregulate CTLA-4 and hinder T cells from progressing through the cell cycle, with the effect more prominent in naïve T cells compared to those already antigen-activated.^38^ Interestingly, the CTLA-4 inhibitor ipilimumab was able to reverse the effect to some extent, however, PD-1 blockade had no effect. The influence of corticosteroids on the efficacy of immunotherapies has contradicting results, however, when used at low dose, and administrated after the use of immunotherapy, reduces loss of efficacy.^39^ Notably, dexamethasone is administered at high doses at diagnosis for nearly all children with DMG, and so the use of chimeric antigen receptor (CAR) T cells activated ex vivo, could be an alternative way to avoid the immunosuppressive actions of concurrent corticosteroid therapy.

### DMG Microenvironment

The CNS was historically seen as an immunologically privileged site, with little immune cell infiltration; however, this has been proven to be incorrect as immune cells travel between the CNS and periphery via the choroid plexus, parenchyma, and the leptomeningeal blood vessels.^40^ The brainstem is responsible for coordinating life-sustaining functions such as cardiac and respiratory control and motor function including swallowing, so there exists a delicate balance between effective protection against pathogens (requiring immune surveillance) and the potential harm of excessive inflammation mediated via an immune response. One mechanism the body harbors to avoid excessive inflammation is that of immune checkpoint proteins (discussed in the section “Immune checkpoint inhibitor side effects”). Indeed, a small-cell lung cancer patient treated with the ICI nivolumab (anti-PD-1) developed brainstem encephalitis, highlighting the importance of immune checkpoint proteins in the brainstem.^41^ However, few studies have investigated immune cell infiltration in the healthy brainstem and hence are required to determine the baseline immune microenvironment and its influence throughout the course of the disease. This will reveal whether the cold TME seen in DMG is induced by the tumor itself, or if the lack of immune cells is the native state of the brainstem.

Analysis of the TME of DMG showed a dramatic reduction in the residency of immune cells compared to normal brain tissue, as well as a lack of chemokines necessary for recruitment of immune cells (Figure 1).^10^ The expression of the T-cell marker CD3 is substantially lower in DMG tissues compared to adult glioblastoma (aGBM) as well as other pediatric HGG and LGG. The lack of CD3^+^ lymphocytes in DMG could potentially be explained by failed recruitment, as DMG release considerably fewer cytokines and chemokines than aGBM.^42^ Consistent with the low number of CD3^+^ cells, RNA sequencing, and immunohistochemistry of DMG autopsies showed that the majority of DMG have few, if any, infiltrating CD8^+^ T cells.^10,43^ In contrast, primary DMG tissue samples have been shown to express increased levels of IBA1^+^ macrophages/microglia compared to normal tissue (cerebral cortex) suggestive of a role for macrophages/microglia in immune avoidance.^42^ When comparing DMG microglial cells to aGBM microglial cells, DMG expresses less inflammatory factors and does not show enrichment of either M1 or M2 associated genes.^42^ Of note, H3.1K27M cells have been shown to promote expression of immunosuppressive factors including CD163, CD206, and PD-L1 in M0 macrophages when co-cultured, while this has not been seen in H3.3K27M DMG. Overall, H3.1K27M DMG appears to display a colder immune phenotype^10^ and thus, a necessary consideration for the development of IO approaches for these patients with DMG.

DMG also does not show an increase in CD163^+^ cells (a marker for immunosuppressive macrophages) compared to non-cancerous brain tissue. This is in contrast with other pediatric HGG^10^ and is again suggestive of DMG harboring a relatively cold TME.

The role of other immunosuppressive markers, such as TIM3, LAG3, and TIGIT has not yet been thoroughly investigated in DMG and could provide important data. The lack of DMG immunosuppressive gene expression profiles highlights a promising opportunity to induce CAR T cells that are already activated ex vivo and may not be impeded by intrinsic mechanisms or by macrophages/microglia. However, critical differences in the TME across DMG subtypes require further interrogation if immunological approaches are to be successfully studied and implemented.

### Blood-Brain Barrier

The blood-brain barrier (BBB) divides precious brain tissue from the systemic circulation. The BBB controls molecular trafficking, prevents entry of toxins, maintains ionic homeostasis, ensures a low protein environment, separates central and peripheral neurotransmitter pools, and regulates immune surveillance and responsiveness to minimal inflammation and cell damage. It is well recognized that brainstem DMG shows reduced BBB permeability compared to identical tumors in the cerebral cortex,^44^ highlighting why chemotherapies have failed to improve outcomes.^45^ Furthermore, the BBB typically allows molecules of 400-500 Da across the protective layer, seeing antibodies (including ICIs) subject to size exclusion. Thus, novel forms of drug delivery are crucial. Recently, the phase I clinical trial BrainChild-01 (NCT03500991), locoregionally delivered HER2-specific CAR T cells into either the tumor cavity or ventricular system of children with recurrent/refractory CNS tumors and demonstrated preliminary feasibility and tolerability. This experience led to the opening of a phase I trial, BrainChild-03 (NCT04185038), delivering B7-H3-specific CAR T cells into the ventricular system of children with DMG, with promising preliminary results (discussed in the section “Opportunities in DMG”).

Furthermore, as patients diagnosed with DMG frequently receive radiotherapy, it is important to take into consideration its effect on the BBB. Studies have shown diverse results on how radiation manipulates the BBB; most studies point at an increase in BBB permeability following radiation.^46–48^ The size of the tumor, the amount of radiation, and timing of concurrent treatment all seem to influence how BBB permeability is altered.^48,49^ This increase in permeability is most likely caused by direct damage of the cells comprising the BBB as well as a subsequent increase in inflammation and oxidative stress. Unfortunately, this increased permeability has not been associated with better delivery of chemotherapies to the CNS, emphasizing the need for further studies elucidating how radiation modifies BBB permeability and its implications in drug delivery. On the other hand, radiotherapy in combination with immunotherapy has been explored in both DMG and other CNS malignancies with positive results (further discussed in the section “Radiotherapy”). Given the epigenetic, somatic, non-inflammatory microenvironment, and impaired adrenal function of patients with DMG, as well as the obstacle that is the BBB, researchers face an uphill battle to develop effective IO treatment strategies that will improve outcomes (Figure 1). Below we provide insights into IO approaches that have transformed the treatment of other cancer types to highlight areas of importance and discuss areas of failure that may influence future investigations focused on developing IO strategies for patients with DMG.

## Immuno-oncology Strategies Targeting Cancer

### Immune Checkpoint Inhibitors

#### PD-1 and PD-L1.

—Tumor cells present tumor-specific antigens through the MHC complex, making them vulnerable to T-cell destruction. However, some cancers have developed methods to escape the immune system via increased expression of inhibitory immune checkpoints, such as programmed cell death ligands 1 and 2 (PD-L1, PD-L2) and cytotoxic T-lymphocyte-associated protein 4 (CTLA-4). PD-1 is expressed on various immune cells including activated T cells. Binding of PD-L1 results in decreased activation, proliferation, cytokine secretion, and cell survival of the T cells.^30^ Several types of cancer, including melanoma, lung, breast, and bladder cancers have been shown to express PD-L1 causing a decline in cytotoxic lymphocytes (CTLs), promoting tumor cell survival. Monoclonal antibodies (mAbs) targeting both PD-1 and PD-L1 have been proven to be effective in several cancers. This includes PD-1 inhibitors, such as nivolumab, pembrolizumab, cemiplimab and PD-L1 inhibitors, such as atezolizumab, avelumab, and durvalumab, all FDA-approved.^30^ Currently, several clinical trials investigating the efficacy and safety of PD-1 mAbs in DMG are ongoing (Table 1); the results so far have been underwhelming (discussed below). No survival benefit was observed in three children with recurrent DMG after treatment with pembrolizumab and one of the participants developed moderate hypersensitivity pneumonitis as a suspected side effect. Additionally, a shorter PFS and rapid neurological deterioration were observed after pembrolizumab treatment in five children with progressive disease in a 2015 study. Importantly, this was reported in an abstract with correlative analysis still ongoing, thus limited information on the cause of the short PFS is available. Interestingly, a retrospective analysis revealed recurrent or refractory CNS tumors treated with either ipilimumab (CTLA-4 mAb), nivolumab, pembrolizumab, or, nivolumab with pembrolizumab, showed no overt toxicity in two pediatric patients with DMG. Nevertheless, both discontinued the treatment after 1.6 and 2.6 months, respectively, due to disease progression. Furthermore, the combination of reirradiation and nivolumab in patients with DMG at disease progression showed no severe toxicities, and a positive trend toward improved OS when compared to reirradiation alone (though not significant).^50^ Lastly, pidilizumab (MDV9300) showed acceptable toxicity profiles in nine children with DMG at diagnosis and post-radiation.^51^ Unfortunately, none of these studies reported PD-1/PD-L1 expression prior to treatment or immune cell infiltration post-treatment. Only one study has investigated the potential of PD-L1 inhibitors in DMG, more specifically durvalumab (NCT02793466), with the results yet to be released. One explanation for the dissatisfying results of PD-1/PD-L1 inhibition could be a lack of expression of the checkpoint proteins in DMG cells. A study evaluating PD-L1 expression in DMG found that none of the cases investigated (n = 31) showed membrane expression of PD-L1. Interestingly, another study found 17.9% of pediatric DMG (n = 28) and 29.4% of adult DMG (n = 34) to be PD-L1-positive, suggesting large heterogeneity between patients and studies.^43^ RNA sequencing of DMG autopsies suggests that the majority of DMG have low expression of PD-L1, however, a subset of these patients displayed a 2-fold increase of PD-1 (54%) and PD-L1 (11%) compared to normal brain tissue.^52^ This is a superb indication of the importance of molecular analysis prior to the treatment of an individual patient. As DMG typically lacks active immune infiltration, blanket use of PD-1/PD-L1 inhibitors for all DMG diagnoses is unlikely to yield substantive improvements to OS but may be of benefit in combination with other targeted treatments. The use of ICI in combination with pro-inflammatory cytokines is yet to show a clear and predictable benefit across different tumor types, however, as the combination of ICI and IL-2 has been deemed safe, there is potential for this approach to be tested in DMG.

**Table 1.** Ongoing Clinical Trials of Immunotherapies in DMG With Status at Submission of This Review

Study Title	Treatment	Type of Therapy	Phase	Status	Start Year	NCT Number
Pembrolizumab in Treating Younger Patients with Recurrent, Progressive, or Refractory High-Grade Gliomas, Diffuse Intrinsic Pontine Gliomas, Hypermutated Brain Tumors, Ependymoma or Medulloblastoma	Pembrolizumab	ICI (anti-PD-1)	I	Recruiting	2015	NCT02359565
Durvalumab in Pediatric and Adolescent Patients	Durvalumab	ICI (anti-PD-L1)	I	Unknown	2016	NCT02793466
An Investigational Immuno-Therapy Study of Nivolumab Monotherapy and Nivolumab in Combination With Ipilimumab in Pediatric Patients With High Grade Primary CNS Malignancies (CheckMate 908)	Nivolumab, Nivolumab + Ipilimumab	ICI (anti-PD-1, anti-CTLA-4)	Ib/II	Active, not recruiting	2017	NCT03130959
Anti PD1 Antibody in Diffuse Intrinsic Pontine Glioma	Pidilizumab	ICI (anti-PD-1)	I/II	Unknown	2013	NCT01952769
REGN2810 in Pediatric Patients with Relapsed, Refractory Solid, or Central Nervous System (CNS) Tumors and Safety and Efficacy of REGN2810 in Combination With Radiotherapy in Pediatric Patients With Newly Diagnosed or Recurrent Glioma	REGN2810/cemiplimab	ICI (anti-PD-1)	I/II	Recruiting	2018	NCT03690869
H3.3K27M Peptide Vaccine with Nivolumab for Children With Newly Diagnosed DIPG and Other Gliomas	Nivolumab + K27M peptide	ICI (anti-PD-1) + peptide vaccine	I/II	Recruiting	2016	NCT02960230
Study of B7-H3-Specific CAR T Cell Locoregional Immunotherapy for Diffuse Intrinsic Pontine Glioma/Diffuse Midline Glioma and Recurrent or Refractory Pediatric Central Nervous System Tumors	B7-H3-specific CAR T-cell locoregional therapy	CAR T-cell therapy	I	Recruiting	2019	NCT04185038
GD2 CAR T Cells in Diffuse Intrinsic Pontine Gliomas (DIPG) & Spinal Diffuse Midline Glioma (DMG)	GD2 CAR T-cell therapy	CAR T-cell therapy	I	Recruiting	2019	NCT04196413
C7R-GD2.CAR T Cells for Patients with GD2-expressing Brain Tumors (GAIL-B)	C7R-GD2 CAR T-cell therapy	CAR T-cell therapy	I	Recruiting	2019	NCT04099797
Immune Modulatory DC Vaccine Against Brain Tumor	Autologous DCs pulsed with genetically modified tumor cells or tumor-related antigens including neoantigens	DC vaccine	I	Enrolling by invitation	2019	NCT03914768
Adjuvant Dendritic Cell Immunotherapy for Pediatric Patients with High-grade Glioma or Diffuse Intrinsic Pontine Glioma (ADDICT-pedGLIO)	WT1 mRNA-loaded autologous monocyte-derived DCs	DC vaccine	I/II	Recruiting	2021	NCT04911621
Precision Medicine and Adoptive Cellular Therapy (PEACH)	Total tumor mRNA-pulsed autologous DCs (TTRNA-DCs), Tumor-specific ex vivo expanded autologous lymphocyte transfer (TTRNA-xALT)	DC vaccine	I	Recruiting	2021	NCT04837547
Brain Stem Gliomas Treated With Adoptive Cellular Therapy During Focal Radiotherapy Recovery Alone or With Dose-intensified Temozolomide (Phase I) (BRAVO)	Total tumor mRNA-pulsed autologous DCs (TTRNA-DCs), Tumor-specific ex vivo expanded autologous lymphocyte transfer (TTRNA-xALT)	DC vaccine	I	Recruiting	2018	NCT03396575
Neoantigen Vaccine Therapy Against H3.3-K27M Diffuse Intrinsic Pontine Glioma (ENACTING)	H3.3K27M targeted neoantigen peptide	Peptide vaccine	I	Recruiting	2021	NCT04749641
rHSC-DIPGVax Plus Checkpoint Blockade for the Treatment of Newly Diagnosed DIPG and DMG	rHSC-DIPGVax + Balstilimab + Zalifrelimab	Peptide vaccine + ICI (anti-PD-1, anti-CTLA-4)	I	Recruiting	2021	NCT04943848
A Pilot Study of SurVaxM in Children Progressive or Relapsed Medulloblastoma, High Grade Glioma, Ependymoma and Newly Diagnosed Diffuse Intrinsic Pontine Glioma	SurVaxM	Peptide vaccine	I	Not yet recruiting	2021	NCT04978727
A MultIceNTER Phase I Peptide VaCcine Trial for the Treatment of H3-Mutated Gliomas (INTERCEPT-H3)	H3K27M peptide vaccine	Peptide vaccine	I	Not yet recruiting	2021	NCT04808245
PEP-CMV Vaccine Targeting CMV Antigen to Treat Newly Diagnosed Pediatric HGG and DIPG and Recurrent Medulloblastoma	PEP-CMV	Peptide vaccine	II	Not yet recruiting	2021	NCT05096481
A Study of Bempegaldesleukin (BEMPEG: NKTR-214) in Combination With Nivolumab in Children, Adolescents and Young Adults With Recurrent or Treatment-resistant Cancer (PIVOT IO 020)	Bempegaldesleukin + nivolumab	IL-2 cytokine prodrug + ICI (anti-PD-1)	I/II	Recruiting	2021	NCT04730349
Pediatric Trial of Indoximod With Chemotherapy and Radiation for Relapsed Brain Tumors or Newly Diagnosed DIPG	Indoximod	IDO pathway inhibitor	II	Recruiting	2019	NCT04049669
Oncolytic Adenovirus, DNX-2401, for Naive Diffuse Intrinsic Pontine Gliomas	DNX-2401	Oncolytic virus	I	Active, not recruiting	2017	NCT03178032
Clinical Trial to Assess the Safety and Efficacy of AloCELYVIR With Newly Diagnosed Diffuse Intrinsic Pontine Glioma (DIPG) in Combination With Radiotherapy or Medulloblastoma in Monotherapy (AloCELYVIR)	AloCELYVIR	Oncolytic virus	I/II	Recruiting	2021	NCT04758533
Wild-Type Reovirus in Combination With Sargramostim in Treating Younger Patients With High-Grade Relapsed or Refractory Brain Tumors	Wild-type Reovirus + Sargramostim	Oncolytic virus	I	Active, not recruiting	2015	NCT02444546

Abbreviations: CAR, chimeric antigen receptor; CTLA-4, cytotoxic T-lymphocyte-associated protein 4; DC, dendritic cells; DMG, diffuse midline glioma; ICI, immune checkpoint inhibitors; IDO, indoleamine 2,3-dioxygenase 1; NCT, National Clinical Trial; PD-1, programmed cell death protein 1; PD-L1, programmed cell death ligand 1; PEP-CMV, peptide-cytomegalovirus.

#### CTLA-4.

**Fig. 2:**
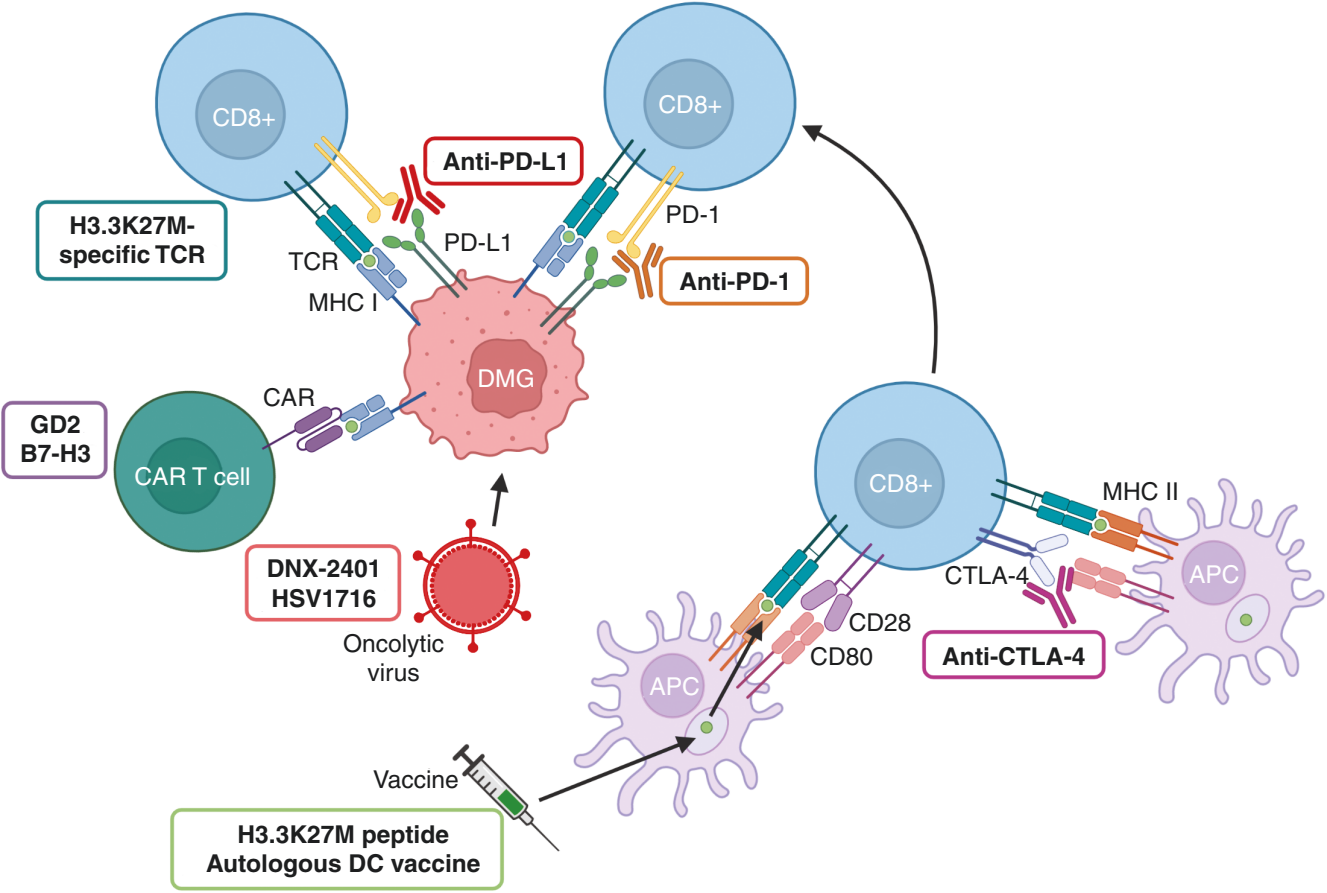
Immunotherapy strategies. Anti-PD-1 and PD-L1 antibodies block the binding of PD-L1 on cancer cells to PD-1 on CD8^+^ T cells impeding the inactivation of CD8^+^ T cells. Anti-CTLA-4 antibodies bind to CTLA-4 on T cells blocking its binding to B7 on APCs allowing B7 to bind to CD28 on T cells resulting in their activation. Vaccines are taken up by APCs and presented to CD8^+^ T cells which become activated and relocate from lymphoid nodes and target the cancer cells. CAR T cells are designed to target DMG cells. Abbreviations: APCs, antigen-presenting cells; CAR T, chimeric antigen receptor T cells; CTLA-4, cytotoxic T-lymphocyte-associated protein 4; PD-1, programmed cell death protein 1; PD-L1, programmed cell death ligand 1.

—The first FDA-approved target for checkpoint blockade was CTLA-4, expressed on naïve T cells. CTLA-4 is a competitive receptor for the B7-1 and -2 ligands which normally bind to CD28, leading to activation and proliferation of T cells.^53,54^ In addition, regulatory T-cells (T_regs_) constitutively express CTLA-4 where, in contrast to other T-cells, it is a critical negative regulator of T-cell responses.^55^ Hence, CTLA-4 blockade maintains effector T-cell activation and proliferation and sustains an anti-tumor response (Figure 2).^55^ Unfortunately, the modest response to PD-1/PD-L1 inhibitors in patients with DMG is mirrored by CTLA-4 antibodies. A retrospective study identified four cases of recurrent or refractory DIPG or other DMG treated with nivolumab and ipilimumab combination therapy.^56^ All patients discontinued the treatment due to progressive disease, although acceptable toxicity profiles were recorded for two patients with DIPG. To the best of our knowledge, there are so far no published studies investigating the safety or efficacy of CTLA-4 inhibitors as monotherapy in patients with DMG. As for PD-L1, RNA sequencing did not reveal an increase in CTLA-4 expression in autopsies compared to normal brain tissue, implying CTLA-4 inhibitors would not provide benefit as DMG monotherapy.^43^

#### Immune checkpoint inhibitor side effects.

—Immune checkpoint proteins are essential to protect tissues from damaging effects of inflammation and are not uniquely expressed on tumor tissue. Thus, toxicities affecting non-tumor tissues following the administration of ICI therapies, normally referred to as “immune-related adverse events” (irAE), are inevitable. One-third of ICI therapy recipients will experience dermatological toxicities: rash, vitiligo, pruritus, and less commonly toxic epidermal necrolysis and vasculitis.^57,58^ Other side effects include toxicities of the gastrointestinal and endocrine systems, as well as cardiac, neurologic, and pulmonary toxicity. Most side effects are efficiently controlled by administrating steroids; however, in some instances, immune-modulatory drugs may be required or ICI may need to be discontinued.

Anti-CTLA-4 treatment is associated with a higher prevalence of side effects and side effects that are more severe compared to both PD-1 and PD-L1 inhibitors. This is most likely caused by the different mechanism of action for each drug (CTLA-4 affects naïve T cells and PD-1/PD-L1 affects active T cells).^59^ Furthermore, combination therapy with CTLA-4 and PD-1/PD-L1 inhibitors is linked to more frequent and more severe toxicities, presumably a result of T cells being targeted at several stages. Importantly, the long-term ICI side effects remain unclear, a notable gap in the context of the developing immune system of pediatric patient populations.

### Vaccines

An alternative IO approach to treating DMG is to harness the immune system by vaccination. By introducing cancer-specific strands of DNA, mRNA, or peptides to the immune system a tumor-specific immune response can be encouraged (Figure 2). Recently, a H3.3K27M synthetic peptide vaccine was developed after the discovery of an HLA-A*02:01-restricted cytotoxic T lymphocyte (CTL) epitope of H3.3K27M.^60^ The safety and efficacy of the vaccine have been investigated in newly diagnosed H3.3K27M^+^ and HLA-A*02:01^+^ patients with either DIPG or other DMG in the PNOC007 study (NCT02960230). No treatment-related grade 4 adverse events were recorded, however, one patient developed meningitis. Investigators could not unequivocally confirm or dispute the latter as being related to the therapy. The treatment resulted in a 12-month OS of 44% for patients with DIPG and 39% for other DMG.

Antigen-loaded dendritic cells (DC) have also been used as a vaccine. Autologous or allogeneic DCs are isolated, pulsed with the intended antigen/s, and then administered to the patient. The method has been shown to induce tumor-specific immune response and the reported side effects are usually mild and transient.^61^ However, distinct clinical benefits are still to be shown. An autologous DC vaccine pulsed with allogeneic tumoral cell line lysate has been reported to be safe in patients with newly diagnosed DMG.^62^ Additionally, the vaccine was able to initiate a tumor-specific immune response in both peripheral blood mononuclear cells (PBMC) (in 8/9 patients) and cerebrospinal fluid (CSF) (in 2/9 patients), measured by proliferating tumor lysate-specific T cells, suggesting that this could be a favorable approach for treating DMG.^62^ The use of tumor lysate including several subclones or different classes of cancer antigens instead of a single, specific neoantigen as a vaccine could be an approach that limits the risk of immune escape due to downregulation of the target.^63^

### Oncolytic Viruses

Another promising IO strategy for DMG is the use of oncolytic viruses (Figure 2), with several current clinical trials underway (Table 1). Most recently, the adenovirus DNX-2401 (Delta-24-RGD) was shown to be safe in an immunocompetent DMG PDX mouse model, encouraging a phase I clinical trial (NCT03178032).^64^ The virus contains a deletion in the E1A gene, therefore making it selective to cancer cells where the Rb pathway is dysregulated. Additionally, an RGD-motif is added to the viral fiber promoting affinity with α _ν_β _3_ α _ν_β _5_ integrins that are commonly overexpressed on cancer cells, including those of glial nature. The virus showed anti-tumor effects in DMG cell lines in vitro and extended survival of two DMG orthotopic mice models (TP80 and TP45). Interestingly in the mouse model, the administration encouraged CD4^+^ and CD8^+^ T-cell infiltration, determined by mRNA expression, with increased IFN-γ, suggesting recruitment of immune cells, driving viral immunogenic cell death.^65^ Furthermore, the increased survival seen in the immunocompetent DMG mouse model was abolished when the virus was used in immunodeficient PDX models, highlighting the role of the immune response in combination with the viral activity. Excitingly, a case report from the same phase I clinical trial (NCT03178032) illustrated the feasibility of administrating the virus intratumorally at the time of biopsy. The study showed that it was administered safely to an 8-year-old patient with DMG.^66^ The patient left hospital 72 hours after administration with no signs of virus-related toxicities. Four weeks after administration, the patient remained free from drug-related toxicities; however, long-term outcome data are still to be reported. Another oncolytic virus that has been shown to have potential anti-DMG activity is the herpes simplex virus 1716 (HSV1716), which harbors a deletion of ICP34.5 to avoid neurovirulence in healthy cells.^67^ Using a DMG 3D spheroid encased in a collagen matrix, the virus reduces tumor invasion. This was confirmed in a DMG orthotopic mouse model showing a well-contained tumor with very low invasion into surrounding tissue, a notable feat given the typically “diffuse” nature of the disease. It should be highlighted, however, that this was performed in an immunodeficient mouse model and thus a potential benefit of an immune response remains to be shown. Another pressing issue is how to deliver these viruses to such delicate localities. Seeking to address this, an oncolytic adenovirus, CRAd.S.pK7, was encapsulated within mesenchymal stem cells (MSCs) and administered intratumorally to a DMG brainstem xenograft mouse model.^68^ The virus was able to disseminate throughout the tumor, with radiotherapy boosting efficacy. Nevertheless, results from clinical trials investigating oncolytic viruses in DMG are yet to be published; promisingly, preclinical studies and early case reports suggest that viruses are safe and can potentially improve outcomes.

### Adoptive Cell Transfer Strategies

As previously mentioned, DMG displays a low TMB most likely resulting in limited presentation of somatic mutation-derived neoantigens. This, together with the few infiltrating immune cells suggest that ICIs, at least as monotherapy, might not be effective in DMG, echoed by the lack of survival benefit suggested in clinical trials to date. Thus, adoptive cell transfer (ACT), including CAR T cells, which are already activated and designed to target the tumor ex vivo, may constitute a more suitable treatment strategy (Figure 2). Furthermore, ACTs targeting the ganglioside GD2 as well as HER2 show promising preclinical results (discussed below).^69,70^

#### Benefits—ACT success stories.

—Tisagenlecleucel was the first CAR T-cell therapy approved by the FDA in 2017 for the treatment of precursor B-cell acute lymphoblastic leukemia (B-ALL) in children and young adults. Subsequently, the approach has been approved for relapsed or refractory large B-cell lymphoma in adults following two failures with systematic therapies.^71^ Tisagenlecleucel consists of an extracellular domain, a single-chain antibody fragment (scFv), that binds to CD19, which is uniquely and ubiquitously expressed on B cells. The intracellular regions consist of CD3ζ and a co-stimulatory 4-1BB domain, making it a second-generation CAR.^72,73^ The second-generation CAR T-cell therapy to gain FDA approval was axicabtagene ciloleucel (axi-cel) for diffuse large B-cell lymphoma (DLBCL). Analogous to tisagenlecleucel, axi-cel targets CD19, however, it contains a CD28 co-stimulatory domain together with CD3ζ instead of 4-1BB.^74,75^ The most common side effects of both therapies are cytokine release syndrome (CRS) and neurotoxicity, where CRS is managed using IL-6 inhibition (further discussed in the section “CAR T-cell side effects”). Encouragingly for pediatric malignancies, CD19 CAR T cells have also been investigated in children from 1 to 25 years of age with acute lymphoblastic leukemia.^76^ 93% (40 of 43) of patients receiving the CAR T cells achieved minimal residual disease-negative complete remission, with a 12-month OS of 69.5%. Severe CRS and neurotoxicity were recorded in 23% and 21% of patients, but were reversible, supporting its safe use in children. The latest success in CAR T-cell therapy is the approval of idecabtagene vicleucel (ide-cel) for adult relapsed or refractory multiple myeloma, in 2021. Ide-cel targets the B-cell maturation antigen (BCMA), with the most common side effects including CRS and hematologic toxicity.^77^

#### Opportunities in DMG.

—The success of CAR-cell therapies in hematological malignancies encourages investigation in other types of solid tumors. Encouragingly for DMG, CAR T-cell therapy has shown promising results in other CNS malignancies, for instance, glioblastoma.^78^ Several CAR T-cell therapies are currently being investigated for DMG in clinical trials (summarized below) (Table 1).

Screening of surface antigens in four H3K27M DMG patient-derived cell cultures showed high levels of disialoganglioside GD2 expression across the samples.^69^ GD2 expression was confirmed in DMG cultures harboring either *H3F3A* or *HIST1H3B* H3K27M and wild-type H3 HGG, highlighting its potential for patients with DMG. Targeting GD2 with anti-GD2 antibodies is currently being investigated in various other malignancies including neuroblastoma, osteosarcoma, and melanoma.^79–81^ Anti-GD2 therapies kill cancer cells by human complement and other human immune effector cells.^82^ Importantly, activated T cells can cross the BBB, and hence, orthotopic mouse xenografts of patient-derived DMG cell cultures were established and GD2-targeting CAR T cells (containing CD3ζ together with the 4-1BB and CD28 co-stimulatory domains) were intravenously injected into mouse tail veins.^69^ The CAR T cells infiltrated the tumor and reduced the total number of H3K27M^+^ tumor cells compared to treatment with CD19-targeting CAR T cells. However, a small number of H3K27M GD2 negative tumor cells remained, highlighting the heterogeneity of the disease and the need for additional therapies or cellular targets for complete tumor eradication. Positive preclinical results lead to the initiation of recently established human GD2-targeting CAR T-cell therapy clinical trials NCT04196413 and NCT04099797.

The results of four patients with DMG (three patients with DIPG and a H3K27M^+^ spinal cord patient with DMG) treated in the NCT04196413 clinical trial, were recently published.^83^ Encouragingly, three (two DIPG and one DMG) out of the four patients experienced both clinical and radiographical improvements after the first GD2 CAR T-cell infusion administered intravenously post-radiation. The three patients responding received additional infusion intracerebroventricularly resulting in further improvements, increasing survival to 20, 20, and 26 months from diagnosis, respectively. Side effects included CRS, fever, and tumor inflammation-associated neurotoxicity, however, these are managed using tocilizumab, anakinra, and corticosteroids. Of additional note, an Ommaya reservoir was required to monitor intracranial pressure and drain CSF. No on-target, off-tumor toxicities were recorded, indicating that GD2 may be a safe target. Overall, these preliminary results are suggestive of suitable tolerability and potential clinical benefit of GD2 CAR T cells against DMG.

Another interesting point is that a recent study showed that addition of IGF1R/IR inhibitors enhanced the effects of GD2 CAR T-cell therapies. In vitro testing of the IGF1R/IR inhibitor linsitinib together with GD2 CAR T cells on a DMG cell line revealed less T-cell exhaustion and a more immature T-cell phenotype, suggesting more durable T-cell activity. Linsitinib also increased the efficacy of lower doses of CAR T cells in vivo in PDX mouse models, providing an imperative to administer lower doses of CAR T cells to reduce side effects.^84^

A DMG clinical trial investigating the safety and efficacy of B7-H3-specific CAR T cells is ongoing (NCT04185038). B7-H3 (CD276) is expressed on antigen-presenting cells (APCs) and plays an inhibitory role in T-cell regulation. It is upregulated in various types of cancers and implicated in tumor invasion, migration, angiogenesis, and epigenetic modifications.^85^ Several preclinical studies show anti-tumor effects in GBM and other pediatric brain tumors using B7-H3 CAR T cells.^86–88^ Importantly, B7-H3 is overexpressed in DMG cells compared to matched normal tissue, highlighting the potential of B7-H3-targeting CAR T cells for these patients.^89^ Encouragingly, preliminary results from an 18-year-old patient diagnosed with DIPG receiving B7-H3 CAR T cells at disease progression showed no dose-limiting toxicities or CRS. Furthermore, detectable CAR T-cell populations were seen in the CSF with the patient experiencing stable disease at the time of submission.^90^

#### Autologous or allogeneic CAR T cells.

—While discussing the opportunity for CAR T-cell therapy it is important to consider the human leukocyte antigen (HLA) type of the patients. HLA is the most polymorphic gene in the human genome therefore based on the HLA type, peptides binding with high affinity to one HLA type might not bind at all to another HLA type.^91^ Thus, identified neoantigens might not be universally suitable drug targets, and it would instead be optimal to identify HLA peptides that bind with a high affinity to a vast amount of HLA types. Unfortunately, the heterogeneity of HLA types in DMG patients makes this nearly impossible. Additionally, T cells derived from patients will be specific for their own HLA types and thus autologous T cells are commonly utilized for CAR T-cell manufacturing. However, the use of autologous or patient-derived CAR T cells is not always feasible as the manufacturing is costly, time-consuming, and, the patient-derived T cells might be dysfunctional or deficient due to the disease or previous treatments.^92^ An alternative for these patients is allogeneic CAR T cells from healthy donors. However, the use of allogeneic T cells brings us back to the importance of HLA matching of the donor and the patient to avoid graft-versus-host disease (GVHD) or graft rejection. The use of allogeneic T cells thus requires finding a HLA match to express the CAR, which can be time-consuming as well. A promising solution is to further edit the CAR T cells. The cells can be gene-edited to not express the TCR or to express a TCR inhibitor.^93^ In addition to these methods, thorough lymphodepletion of the patient before CAR T-cell infusion could be beneficial to give the CAR T cells a chance to proliferate and kill the tumor cells before the patient’s own immune system responds.^93^ HLA non-dependent CAR T cells would make it possible to manufacture large batches of CAR T cells for large populations of patients, which would both reduce the production cost as well as ensure quick access to treatment.

#### CAR T-cell side effects.

—Most studies investigating CAR T-cell side effects have focused on CD19-specific CAR T cells against hematologic cancers. The most common side effect of CAR T-cell therapy is CRS,^94^ characterized by a massive release of pro-inflammatory cytokines including IL-6, IFN-γ, and TNF-α, resulting in systemic inflammation. CRS ranges from mild cases (presenting with fever, headache, myalgia, or fatigue) to severe experiences involving life-threatening multi-organ failure (including cardiac, neurologic, and pulmonary).^95^ CRS seems to be more common in children, a consideration when administrating CAR T cells to pediatric patients with DMG.^95^ The condition is usually treated with IL-6 inhibitors and corticosteroids. Fortunately, IL-6 inhibition does not impair efficacy.^96^ CAR T-cell-related encephalopathy (CRES), more recently known as immune effector cell-associated neurotoxicity syndrome (ICANS), is another potential side effect of CAR T therapy seen in 40% of patients.^97^ ICANS normally develops simultaneously with or after CRS. Although the exact cause remains uncertain, it is believed to be a result of cytokines and/or CAR T cells entering the CNS, highlighting the importance of extensive monitoring in patients with CNS malignancies treated with CAR T therapy. Unfortunately, tocilizumab is not effective against ICANS, and the recommended treatment is corticosteroids. The severity of both CRS and ICANS is associated with disease burden and dosage.

Hydrocephalus (the accumulation of CSF in the ventricles) was the major side effect related to the use of GD2-specific CAR T-cell therapies in DMG PDX mouse models.^69^ However, histological analysis of brains of mice treated with CAR T cells showed a distinct lack of neuron cell death. Hence, CAR T-cell–related toxicities in DMG models were linked to increased neuroinflammation and hydrocephalus caused compression of the fourth ventricle during tumor clearance, and not a consequence of on-target, off-tumor cell death. It is however uncertain if this phenomenon is GD2 specific or a response to CAR T cells in general. Hopefully, this will become clear with the multiple CNS CAR T-cell trials currently ongoing (HER2, EGFR, B7-H3, IL13ra2, etc.).

### Engineering T cells

TCR T-cell therapy is based on the isolation and modification of high-affinity TCR clones transduced into T cells. The first TCR therapy to receive FDA approval was tebentafusp-tebn. Tebentafusp-tebn was approved for unresectable or metastatic uveal melanoma after a phase III clinical trial showed a 12-month OS of 73% compared to 59% in the control group receiving either pembrolizumab, ipilimumab, or dacarbazine.^98^ Importantly, H3.3K27M-mutant peptides bind with high affinity to HLA-A*02:01 to induce CD8^+^ T-cell response in PBMC from HLA-A*02:01 patients with DMG (n = 3). Furthermore, T cells transduced with a H3.3K27M-specific TCR resulted in reduced tumor progression in H3.3K27M^+^ PDX mouse models, proposing H3.3K27M-specific TCR transduced T cells as a potential future treatment.^99^

## Combination Therapy

### Immune Checkpoint Inhibitors and CAR T Cells

Like all other T cells, CAR T cells are vulnerable to inhibition by PD-1/PD-L1 and CTLA-4, thus, several studies have explored the combination of CAR T therapy with ICIs. As previously mentioned, DMG display low expression levels of immune checkpoint proteins, however, studies suggest CAR T-cell administration might cause an upregulation of these proteins, even though this is yet to be investigated in DMG.^100,101^ On a positive note, combining CAR T therapy with PD-1 inhibition has shown positive results in GBM. PD-1 inhibition together with anti-HER2 CAR T cells enhanced the efficacy of the CAR T therapy in a GBM cell line.^99^ PD-1 inhibition combined with anti-EGFRvIII CAR T cells overcame T-cell exhaustion leading to enhanced anti-tumor activity in mice.^100,102^ This has led to clinical trials investigating the safety and efficacy of ICIs with CAR T cells in GBM, however, the results are still to be published. Notably, CAR T cells also express PD-1, making them sensitive to PD-L1 inhibition by cancer cells. Recently, CRISPR-Cas9 disruption of PD-1 on EGFRvIII CAR T cells led to more durable response and longer survival in a mouse model of glioma compared to traditional EGFRvIII CAR T cells.^103^

### Chemotherapy

To enhance the effect of immunotherapies, a possible strategy is to combine them with other more traditional therapies. Chemotherapies alone have not contributed to increased survival for patients with DMG, however, studies show that chemotherapy, notably temozolomide (TMZ) has immune-modulatory effects in GBM, suggesting an opportunity for it to be combined with immunotherapies. TMZ has previously been tested together with both cancer vaccines and CAR T therapy with positive results in GBM.^104,105^ The positive effect is most likely caused by TMZ-induced lymphodepletion prior to the administered immunotherapy. Lymphopenia of resident T cells allows for better expansion of CAR T cells, clears immunosuppressive myeloid cells and lymphocytes, and can induce the expression of inflammatory cytokines, creating a better environment for CAR T-cell activity.^106^ It is uncertain whether the same effect will be seen in DMG after TMZ treatment since TMZ has been unable to induce a clinical benefit in DMG.^45^ Thus, whether the lymphodepletion induced by TMZ is seen in DMG or if it is specific to GBM remains to be investigated. Furthermore, decreased expression of PD-L1 after TMZ administration has also been observed in GBM, preventing the cancer cells from inhibiting T cells. However, this does suggest that chemotherapy and ICI might not work synergistically, and chemotherapy is better paired with CAR T therapy.^107^ To the best of our knowledge, there have been no studies that have investigated the combination of chemotherapies with immunotherapy in DMG. Although studies in GBM suggest the approach may have merit, with chemotherapies yet to show benefit in DMG it may be preferable to investigate alternative combinations as a priority.

### Radiotherapy

As previously mentioned, radiation can break down the BBB, thus allowing therapies to penetrate and reach the CNS. Furthermore, the radiation-induced cell death causes release of cancer antigens encouraging an immune response that may enhance the efficacy of immunotherapies. Favorably, a retrospective cohort analysis comparing patients with DIPG treated with either nivolumab alone, or together with reirradiation, revealed the combination to be well tolerated and to prolong OS slightly from 20.4 to 22.9 months compared to reirradiation alone. Although not significant (*P* = .44), the trend “points toward a potential improvement”.^50^ The combination has also shown positive results in GBM. Stereotactic radiation together with PD-1 mAb in a mouse model resulted in long-term survival in 15%-40% (>180 days), an effect not seen in mice treated with either radiation or PD-1 blockade alone.^108^ Likewise, the combination of radiotherapy, IDO inhibition, and PD-1 inhibition has been shown to increase survival in mice, further supporting the combination of radiotherapy with ICI.^109^ Furthermore, the addition of radiotherapy prior to administering pembrolizumab in NSCLC increased OS from 7.6 to 15.9 months in the phase II clinical trial PEMBRO-RT (NCT02492568).^110^ In addition to ICI, radiotherapy has been shown to work synergistically with CAR T therapy in preclinical models of GBM. Mice treated with CAR T cells targeting NKG2D together with radiotherapy survived longer than mice treated with either the CAR T cells or radiotherapy alone.^111^ Likewise, combining CAR T cells against GD2 with radiotherapy led to increased survival compared to GD2 CAR T cells alone.^112^ Studies investigating the combination in DMG are yet to be conducted, highlighting an opportunity to determine whether radiotherapy can enhance the efficacy of IO therapies in DMG.

## Conclusion

Despite 50 years of research, survival for patients diagnosed with DIPG remains just 9-11 months. Patients diagnosed with all forms of DMG are told “there are no treatments.” IO strategies have shown great promise in extending survival for cancers of other origins, but similar benefit is yet to be realized in the DMG setting. The unique epigenetic landscape, inter- and intra-somatic heterogeneity, and immunologically cold TME of DMG, highlight areas of research that require focused attention if we are to exploit the potential of immunotherapeutic approaches for patients with DMG. We believe that although limited response to ICI has been seen thus far, combinations with precision therapies may prove to increase response rates. Furthermore, introducing active immune cells with a specific target to the vicinity of the tumor using ACT is proving to be more beneficial at the current time. Accordingly, CAR T cells, as well as vaccines and oncolytic viruses show promising early-stage results. However, there remains a considerable knowledge gap regarding the immune microenvironment of DMG, hampering the development of successful strategies for patients presently fighting DMG. Future work focused on elucidating the underpinnings of the cold immunological response in DMG is desperately necessary, as well as investigations to reveal the potential expression of other (targetable) immune checkpoints. The role H3K27M plays in immunosuppression, and the potential for immunopeptidomics studies using biopsy samples may arm us with novel CAR T-cell therapies that show greater efficacy and specificity. Finally, assessing what roles the various combinations of driver and passenger mutations (including germline) play in the DMG-immune axis is critical. These data will provide us with the potential to co-target these mutations in combination with immunotherapies, to improve response rates, and to better inform which patients will benefit from these sophisticated regimens. Harnessing this information in the development of combination treatment modalities is necessary if we are to improve the likelihood of achieving long-term patient survival for children and young adults diagnosed with DMG.
